# Klatskin-Mimicking Lesions

**DOI:** 10.3390/diagnostics11111944

**Published:** 2021-10-20

**Authors:** Marinko Marušić, Matej Paić, Mia Knobloch, Marko Vodanović

**Affiliations:** 1 Department of Hepatogastroenterology, University Hospital Sveti Duh, 10000 Zagreb, Croatia; marusic1959@gmail.com (M.M.); matejpaic0@gmail.com (M.P.); markovod@gmail.com (M.V.); 2 Faculty of Medicine, J.J. Strossmayer University of Osijek, 31000 Osijek, Croatia; 3 Faculty of Health Studies, University of Rijeka, 51000 Rijeka, Croatia

**Keywords:** hepatic malignancy, cholangiocarcinoma, Klatskin-mimicking, Klatskin-like

## Abstract

Altemeier-Klatskin tumor is a perihilar cholangiocarcinoma that occurs within 2 cm of the confluence of the right and left hepatic duct at the hepatic hilum and accounts for 50–70% of all cholangiocarcinomas cases. Although imaging techniques have come very far today, this entity can still be very challenging to diagnose as there are many lesions that can mimic Klatskin tumor. In this review, we will present the most common Klatskin-mimicking lesions.

## 1. Introduction

Hepatocellular carcinoma (HCC) is the most common primary hepatic malignancy and the second most common is cholangiocarcinoma (CCA) comprising 10–25% of primary hepatic malignancies [1,2]. The location of CCA defines three different subtypes: intrahepatic (iCCA), perihilar (pCCA) and distal (dCCA).

Altemeier-Klatskin tumor is perihilar/hilar CCA that occurs within 2 cm of the confluence of the right and left hepatic duct at the hepatic hilum and accounts for 50–70% of all CCA [2]. Hilar CCA has three morphological types: the most common is periductal infiltrative (70%), polypoid and exophytic, depending on the predominant pattern of spread in relation to the duct wall. 

Hilar CCA occurs most commonly during the sixth decade of life [3]. The diagnosis can be challenging and requires a high suspicion in order to detect lesions that mimic Klatskin tumor but are not malignant and do not require wide resection. 

CCAs are usually asymptomatic in early stages and therefore, often diagnosed when the disease is already in advanced stages [4]. Unfortunately, both malignant and benign biliary strictures share the same clinical symptoms and signs—jaundice, pruritus, dark urine, clay-colored stool, abdominal discomfort, weakness. Bilirubin level and serum tumor markers such as carbohydrate antigen 19-9(CA19-9), interleukin-6 (IL-6) and neutrophil gelatinase-associated lipocalin do not have the power to reliably differentiate malignant from benign stricture [5]. Whereas painless progressive jaundice with anorexia and weight loss favors malignancy and previous biliary surgery favors benignity [6]. 

**Figure 1:**
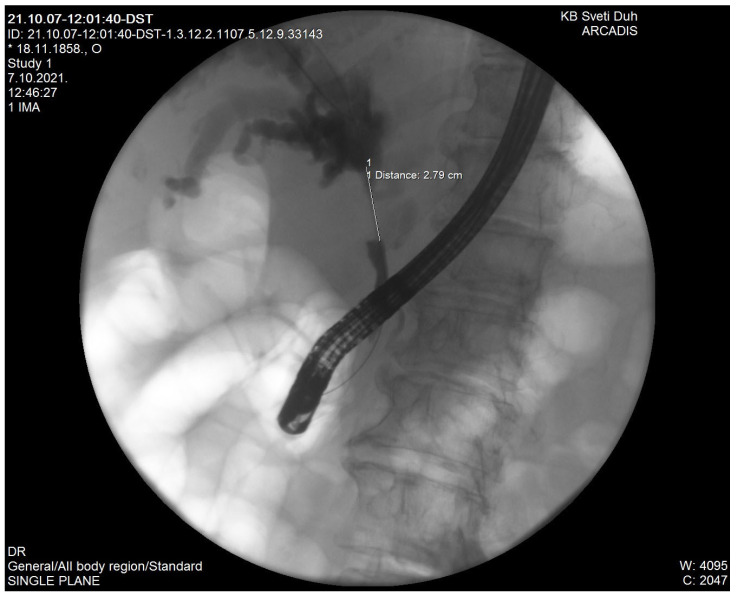
Klatskin tumor on ERCP (ownership of the author).

Ultrasound (US), computed tomography (CT) and magnetic resonance imaging (MRI) are used for CCA detection. CT scanning plays a key role in the diagnosis of hilar CCA which appears on CT as a hyperattenuating intraductal mass, focal mural thickening or lumen obliteration at the hilar bile duct. It is possible to conduct CT-guided percutaneous biopsy (fine-needle aspiration or core needle biopsy) of the hilar lesion to preoperatively establish a definite tissue diagnosis but currently it is recommended to avoid biopsy of presumed hilar CCA because of the risk of complications such as tumor seeding or bleeding [7]. MRI in combination with magnetic resonance cholangiopancreatography (MRCP) is excellent for the diagnosis of hilar CCA. Sensitivity and specificity for the detection of bile duct malignancy are 81–100%, respectively [8]. The role of positron emission tomography/computed tomography (PET/CT) remains unclear in the differential diagnosis of hilar strictures [9]. Percutaneous transhepatic cholangiography (PTC) and endoscopic retrograde cholangiopancreatography (ERCP) are commonly used for differential diagnoses of biliary strictures at the liver hilum but a significant limitation is their invasiveness and their failure to depict the whole biliary tree in the event of complete biliary obstruction (Figure 1). On the other hand, these methods are good for biliary drainage and sampling for brush cytology and forceps biopsy, although cytology and biopsy have low sensitivity for hilar CCA (around 50%) since cancerous tissue is mainly located in the fibrous stroma surrounding the bile ducts [10]. In patients with highly presumed hilar CCA but negative cytology and biopsy, it is possible to conduct an endoscopic ultrasound (EUS)-guided FNA (fine needle aspiration) with a sensitivity of 77–89% and specificity of 100% for the diagnosis of hilar CCA [11]. However, as mentioned by CT-guided percutaneous biopsy, since there is a significant concern over tumor seeding by FNA it is currently recommended to avoid EUS-guided FNA in a presumed surgically curable hilar CCA [7].

According to a recent large series of patients with hilar strictures and preoperative diagnosis of hilar CCA, in 16% of cases, postoperative diagnosis proved to be a benign lesion [12,13,14,15,16]. In this paper, we will focus on other lesions that can masquerade Klatskin tumors as there are a plethora of similar lesions, and despite sophisticated imaging techniques we have available today, hilar CCA is still challenging to accurately diagnose preoperatively (Table 1). It is of great importance to make the right diagnosis, especially keeping in mind that benign conditions usually do not require major surgical interventions and can be treated with percutaneous balloon dilatation or endoscopic or percutaneous stent placement [17].

## 2. Recurrent Pyogenic Cholangitis

Recurrent pyogenic cholangitis or oriental cholangiohepatitis is a condition of recurrent infections of the biliary tree associated with intrahepatic strictures and hepatolithiasis endemic to South East Asia. It is characterized by the presence of multiple intraductal calculi causing subsequent biliary tree dilatation and stricture without extrahepatic biliary obstruction [18]. Typical clinical manifestations are Charcot’s triad (recurrent fever, right upper quadrant pain and jaundice) or Reynold’s pentad (Charcot’s triad + hypotension and altered sensorium). Chronic infections with common helminths such as Clonorchis sinensis and Ascaris lumbricoides are thought to induce biliary epithelial inflammation, reduce host immune response and subsequently increase susceptibility to ascending infection from translocating gut pathogens such as *E. coli*, *Proteus*, and *Klebsiella* [19,20]. In summary, recurrent pyogenic cholangitis occurs due to hepatolithiasis, which stimulates a vicious cycle of recurrent bacterial infection, chronic epithelial damage and ongoing biliary inflammation [18]. Possible hilar stricture due to hepatolithiasis and chronic epithelial damage can be misinterpreted as hilar CCA. The best method to define calculi, the extent of stricture and potential lobar atrophy is MRI.

## 3. Mirizzi Syndrome

Mirizzi syndrome is the obstruction of the common hepatic duct by a gallstone impacted in the cystic duct or Hartmann’s pouch. Clinical presentation of the syndrome can be from no symptoms to severe acute cholangitis with inflammation around the hilum which may result in stricture and the stricture can mimic hilar CCA [21]. Gallstones are not always seen on a CT so the method of choice for identification of calculi and extrinsic compression is MR cholangiopancreatography.

## 4. Stricture in Primary Sclerosing Cholangitis

Primary sclerosing cholangitis (PSC) is a disorder of unknown etiology, characterized by a progressive inflammatory, sclerosing and obliterative process of the extrahepatic or/and intrahepatic bile ducts [22]. Patients are often asymptomatic at the time of diagnosis, but the disease is suspected in otherwise unexplained abnormal elevation in serum alkaline phosphatase (4–10 times normal) and serum aminotransferase usually 300 IU/L. The diagnosis is usually established by MRCP showing multifocal stricturing with normal and/or dilated areas of intrahepatic or extrahepatic bile ducts. Moreover, although PSC is characterized by a beaded appearance on imaging, a dominant stricture at the hilum may be mistaken for a CCA. Brush cytology may facilitate the diagnosis, however, cytologic evaluation along with MRI or CT and serum tumor markers (CEA and CA 19-9) is not uniformly helpful in ruling out the diagnosis of CCA. 

## 5. Portal Hypertensive Biliopathy

Portal hypertensive biliopathy is a term referring to changes in the entire biliary tract including intrahepatic and extrahepatic bile ducts, cystic duct and gallbladder in patients with portal hypertension. It is caused by portal hypertension and ischemia of the biliary tree from the collateral veins that evolve in portal hypertension. It is seen more often in patients with extrahepatic portal venous obstruction (80%) than in patients with cirrhosis, where the frequency is up to 30%. The mechanism of ischemia resulting in bile duct damage in patients with extrahepatic portal venous obstruction is still unclear. Investigation of choice in suspected portal hypertensive biliopathy is MR cholangiography that shows typical smooth strictures and segmental dilatations. Possible stricture at the hilum may mimic cholangiocarcinoma [23].

## 6. Heterotopic Tissue

In extremely rare cases, a heterotopic tissue can appear in the bile duct. Only a few cases have reported heterotopic tissue of gastric and pancreatic cells and so far, there was only one case report of chondroid tissue. Moreover, although it is a benign condition, it should be considered in the differential diagnosis of stricture and mass-forming lesions of the bile duct [24].

## 7. Ischemic Cholangiopathy

Bile ducts are supplied only by the hepatic artery so ischemic cholangiopathy can occur as a complication after liver transplantation. During liver transplantation, usually five anastomoses are made, one of them is hepatic artery anastomosis. Stenosis or thrombosis of the hepatic artery may occur as a complication after liver transplantation,. Depending on the extent of the arterial obstruction, ischemic cholangiopathy may present as acute or chronic disease. If acute, it can present as a formation of the biliary cast and if chronic it may mimic primary sclerosing cholangitis or cholangiocarcinoma. Stenosis in chronic ischemic cholangiopathy is predominantly seen at the middle third of the common bile duct and hepatic confluence. Diagnosis is suspected if the patient has a history of liver transplantation, jaundice and hepatic artery anomaly on Doppler ultrasound but otherwise it can mimic Klatskin tumor [25].

## 8. Inflammatory-Infiltrative

### 8.1. Inflammatory Pseudotumor

Inflammatory pseudotumor (IPT) are quite rare and benign lesions that can be found throughout the body, most commonly affecting the lung and orbit, but also in the stomach, liver, spleen, biliary tract, mesentery, urinary bladder, kidneys and other sites. They are called pseudotumors as they often mimic malignant lesions, despite being benign in character. Moreover, histologically, they consist of inflammatory cells (lymphocytes, plasma cells, myofibroblastic spindle cells, and collagen) [26], with an unknown etiology. On rare occasions, IPT can be found in the biliary tract, where they can resemble hilar carcinoma. Some are associated with PSC, recurrent pyogenic cholangitis, Crohn’s disease, and IgG4 related sclerosing disease with or without pancreatitis [27,28,29]. Imaging techniques are not specific but biliary lesions show focal mass with delayed portal venous enhancement due to fibrous component, which is a feature similar to CCA, therefore radiologically it is hard—if at all possible—to differentiate them before laparotomy. IPT can regress spontaneously but symptomatic lesions causing biliary obstruction may need surgical resection or endoscopic treatment. 

### 8.2. IgG4 Sclerosing Cholangitis

IgG4 sclerosing cholangitis (IgG4 SC) is a manifestation of IgG4 related disease which is an inflammatory condition affecting multiple organs. IgG4 autoimmune pancreatitis is a prototype of IgG4 related disease, but other manifestations can be seen, comprising the aforementioned IgG4-related sclerosing cholangitis, sclerosing sialadenitis, orbital disease and retroperitoneal fibrosis. IgG4 SC can be found in up to 70–90% of patients with autoimmune pancreatitis [30,31]. The etiology of this condition is not fully understood but it is believed to be of autoimmune origin. Patients present with nonspecific symptoms, mostly biliary obstruction, weight loss and mild abdominal pain, with typical male predomination around the sixth decade of life. Diagnosis is easily obtained if surgical pathohistological specimens are available, as plasma cells dominate on immunostaining with storiform fibrosis, lymphoplasmacytic infiltration, obliterative phlebitis, and eosinophilic infiltration. High levels of serum IgG4 are also specific for IgG4 SC, but not always positive. Imagining modalities can show stenotic bile duct segments with thickened walls but this condition is difficult to differentiate from PSC and CCA relying on imaging alone. Computerized tomography can, however, indirectly detect autoimmune pancreatitis associated with this condition, which could point to this condition as they often coexist. Brush cytology is unfortunately not reliable and of low sensitivity. Treatment consists of corticosteroid therapy, although in some cases steroid-refractory disease requires anti-TNF-alpha treatment. 

### 8.3. Eosinophilic Cholangiopathy

Eosinophilic cholangiopathy (EC) is an extremely rare condition with so far only sporadically published case reports, first reported in 1985 [32]. It is a part of a larger spectrum with eosinophilic infiltration of other organs, and predominantly, but not always necessary, peripheral eosinophilia. Eosinophilic infiltration is more commonly seen affecting the gallbladder, in eosinophilic cholecystitis, but kidneys, urethra, and stomach can also be involved. Ductal infiltration with stenotic segments leading to jaundice, rarely with mass lesions, is typical which can be difficult to set apart from CCA or PSC. Diagnosis is easy to establish if there are histological specimens available, but generally, that is not the case. There are no specific imagining characteristics to confirm this diagnosis and surgery remains the only option if malignancy cannot be excluded. However peripheral eosinophilia, good response to corticosteroid therapy, reversal of biliary abnormalities even without treatment and other organ involvement (pancreas, gastrointestinal, bone marrow) can be of help.

### 8.4. Mast Cell Cholangiopathy and Follicular Cholangitis

Mast cell cholangiopathy and follicular cholangitis are extremely rare cases of biliary tract obstruction and only a few cases have been reported so far [33,34,35,36,37]. 

### 8.5. Xanthogranulomatous Cholangitis (XGC)

XGC is a rare condition that can be seen in xanthogranulomatous cholecystitis, an uncommon cause of chronic infiltrative cholecystitis that can mimic gallbladder carcinoma. Xanthogranulomatous cholecystitis shows local aggressive behavior and can spread and infiltrate local organs and is usually related to gallbladder lithiasis [38]. If extrahepatic bile ducts are involved, particularly of the hilum, this condition is difficult to separate from CCA based on imaging alone. Surgical resection can confirm the diagnosis. 

### 8.6. Sarcoidosis

This is a chronic, multisystemic condition marked by noncaseating granulomas, typically found in younger women between ages 20–40, most commonly involving the lungs [39] but also affecting extrathoracic lymph nodes, skin, eyes, and hepatobiliary system. Liver involvement rarely manifests clinically but is seen in histopathological samples in 50–70% of the patients with sarcoidosis [40]. If symptomatic, it can present with hepatomegaly, right upper quadrant pain or elevated liver enzymes. The longstanding disease can lead to fibrosis and eventually even liver cirrhosis requiring transplantation. Extrahepatic biliary manifestations are due to strictures of the bile ducts or lymph node involvement, which can imitate CCA if hilar lymph nodes are affected [41]. Targeted biopsies are needed to confirm the diagnosis which can be further supported with other typical organ involvement, high levels of angiotensin-converting enzyme and hypercalcemia. Strictures causing jaundice require drainage (surgical or endoscopic), but other treatment options such as steroid therapy or ursodeoxycholic acid showed to benefit cholestasis.

## 9. Infective

### 9.1. Tuberculosis

Tuberculosis (TB) of the biliary tree, seen in abdominal TB is an extremely rare entity, although needs to be kept in mind in patients with confirmed TB or past history of TB. The biliary tree can be affected due to ascending infection via the ampulla of Vater, hematogenous spread or descending infection through secretion of the organisms in the bile and distally to the bile ducts. It needs to be suspected in patients with other symptoms of TB, longstanding low fever, anorexia, abdominal pain, and other organ involvement, notably the lungs. In biliary tree TB, symptoms are due to either lymph node compression [42], or primary bile duct TB that can cause strictures [43]. Imaging studies are not specific so differentiation from CCA can be difficult. Diagnosis is made histologically or with bacteriological evidence of infection and needs to be suspected especially in endemic or high prevalence areas. Besides antituberculous pharmacotherapy, some cases will require specific treatment pertaining to jaundice resolution. 

### 9.2. AIDS Cholangiopathy

It was first described and published in 1986 [44] when it was seen in patients with acquired immunodeficiency syndrome (AIDS) related opportunistic infections of the biliary tree who presented with biliary obstruction. Since then, there have been numerous reports of this condition. It is usually seen in the later stages of human immunodeficiency virus (HIV) disease with developed AIDS and the most common causes of AIDS cholangiopathy are opportunistic infection with Cytomegalovirus (CMV) and Cryptosporidium parvum. It can present with jaundice, right upper quadrant abdominal pain and elevated liver enzymes. It needs to be kept in mind in AIDS patients who present with those symptoms. Ultrasound can verify common bile duct dilatation with distal stenosis or thickened wall of the bile duct. ERCP is the method of choice where several types of AIDS cholangiopathy are recognized—sclerosing cholangitis, with or without papillary stenosis, and long extrahepatic bile ducts strictures [45]. If the latter is seen in the hilar part of bile ducts, then cholangiocarcinoma resemblance can be an issue. Opportunistic infection treatments have not proved to be very effective in resolving AIDS-related cholangiopathy and endoscopic procedures may be needed. 

### 9.3. Other Infective Causes

Limited to sporadic cases some bacterial infections can cause Klatskin-like lesions, such as *Escherichia coli* O157 associated colitis with secondary sclerosing cholangitis [46], Actinomycosis involving the gall bladder or the bile ducts [47], and other severe systemic infections. The etiology of the latter is not fully understood but cholestasis seems to be the common factor that usually resolves with the resolution of the infection, nevertheless, there have been cases where it doesn’t subside and can lead to secondary sclerosing cholangitis [48]. Some fungal infections have been described to cause mass lesions and hilar strictures such as mucormycosis [49] as well as already described parasitic infection with *Clonorchis sinensis* [50] and *Ascaris lumbricoides* causing recurrent pyogenic cholangitis.

## 10. Benign Tumors

Benign tumors of the extrahepatic biliary tree are rare and include papillomas, polyps, adenomas, fibromas, neurinomas, leiomyomas, hamartomas, granular cell myoblastomas, lipomas, granulomas, carcinoids, and adenomyomas [51]. They account for only 6% of all biliary tumors and 0.1% of all biliary tract operations [52]. Benign tumors usually develop from the epithelial or nonepithelial layers of the bile ducts and exhibit a spectrum of clinical, pathologic and radiologic variants [53]. Even though they are rare, they should always be considered in the differential diagnosis of biliary strictures [54]. Benign biliary tumors generally manifest similar clinical symptoms and signs as malignant except anorexia and weight loss, which are uncommon in patients with benign lesions [14].

### 10.1. Extrahepatic Biliary Adenomas

Extrahepatic biliary adenomas arise from the glandular epithelial cells of the bile ducts, representing the most common type of benign tumors of the extrahepatic biliary tree. In the majority of patients with symptomatic biliary adenomas, obstructive jaundice comprises the prevalent presenting clinical sign [55]. Obstruction of the biliary tree is frequently transitory and can be difficult to differentiate from biliary lithiasis. Biliary adenomas are usually localized in the distal part of the common bile duct, with lesions in the common hepatic duct accounting for only 15% [56]. ERCP with multiple tissue biopsies may be used for obtaining the correct diagnosis [57]. Lesion resection and pathohistological verification are still the most reliable ways of ruling out malignancy. Hence, bile duct resection should be planned for any bile duct stricture when malignancy cannot be ruled out completely [50].

### 10.2. Granular Cell Tumors

Granular cell tumors are uncommon, but when they do occur, it is usually in the oral cavity, skin or subcutaneous tissue. The affection of the biliary tree is sporadic. Differential diagnosis of the hilar granular cell tumors at preoperative examination is difficult and includes CCA, sclerosing cholangitis, or more common benign biliary tumors [58]. Repeated biopsies may be used to obtain a definite preoperative diagnosis. Histopathologically, they consist of large, granular-like eosinophilic cells which are often immunoreactive for the S-100 protein [59]. Treatment consists of surgical excision after which prognosis is favorable.

### 10.3. Neurofibroma

Neurofibromas are uncommon benign tumors arising from Schwann cells. Schwann cells form the myelin sheath of peripheral nerve fibers. They usually develop in the context of neurofibromatosis type I also called von Recklinghausen’s disease. That is an autosomal dominant disorder associated with multiple tumors of the nervous system and can include skin lesions. Only a limited number of extrahepatic bile duct neurofibroma cases have been reported in the medical literature [60,61,62]. These tumors grow on the connective tissue nerve sheath of the vegetative nerve fibers innervating the bile ducts. A definite preoperative diagnosis is extremely difficult. Exploratory laparotomy is usually the procedure of choice, and the type and extent of the procedure should be decided on based upon intraoperative frozen section examination [53].

### 10.4. Schwannoma

Schwannoma or neurilemoma is a benign tumor derived from Schwann cells, which form the inner portion of the peripheral nerve sheaths. Peripheral nerve schwannomas can occur in association with neurofibromatosis type I and II [63]. Digestive tract schwannomas are infrequent, usually arising in the stomach, large bowel, rectum and esophagus [64]. Rarely, these tumors can develop along the vegetative nerve fibers overlying the biliary duct wall [65]. Clinical symptoms and signs are not specific and there are no imaging findings or parameters typical for these tumors [66]. Even when a preoperative suspicion exists, a definitive diagnosis of this exceedingly rare extrahepatic biliary tumor requires histopathological confirmation. Thus, most surgeons agree that surgical resection is the treatment of choice. Digestive tract schwannomas have an excellent prognosis upon adequate surgical management [67].

## 11. Malignant Tumors

### 11.1. Intraductal Papillary Neoplasms of the Bile Duct

Intraductal papillary neoplasms of the bile duct (IPNB) is a recently recognized rare type of epithelial bile duct tumor. It is typically exophytic, exhibiting either a papillary or villous growth within the bile duct lumen. These tumors represent the biliary counterpart of the intraductal papillary mucinous neoplasm of the pancreas (IPMN). Based on these microscopic features, IPNBs are considered as pre-malignant lesions towards invasive CCA. Prior to IPNB inclusion into the WHO classification of tumors, various terminology has been used to describe the spectrum of these tumors, e.g., biliary papilloma/papillomatosis, papillary carcinoma of the extrahepatic bile duct, and biliary cystadenoma and cystadenocarcinoma [53]. Despite the fact that IPNBs are considered as premalignant lesions, their management should be similar to that applied for CCAs, meaning that radical surgical intervention is required because preoperative diagnosis usually underestimates the degree of tumor cell atypia [68].

### 11.2. Neuroendocrine Tumors

Neuroendocrine carcinomas represent a heterogeneous tumor entity, which can occur in almost all organ systems of the human body. The global incidence is low: approximately 0.5–5/100,000. About 70% of NETs occur in the digestive system and about 20% affect the bronchopulmonary tract [69]. In 1996, the WHO proposed the term “neuroendocrine tumors”, abandoning the previously used term “carcinoid”. According to the WHO, the term NET includes all endocrine tumors, ranging from well-differentiated carcinoid tumors to poorly differentiated cancers [70]. The level of proliferation, which defines the tumor grading, is the most important prognostic factor. Overall, poorly differentiated NETs are aggressive tumors bearing a poor prognosis [69]. Most biliary NETs are non-functioning and do not manifest systemic endocrine symptoms [71]. Neuroendocrine tumors of the bile duct are rare and account for 0.2–2% of all gastrointestinal neuroendocrine tumors. Most often they present with jaundice and the most common localization in the biliary tract is the common bile duct. Similar to other tumors of the biliary tree, these lesions are almost impossible to differentiate from CCA [72]. Imaging techniques such as ultrasound, CT or MRI-MRCP alone cannot confirm biliary NETs because they are not specific enough. Definite preoperative diagnosis of extra-hepatic biliary NETs is difficult. The diagnosis is usually confirmed postoperatively by the histopathological analysis of the surgically taken specimen. Occasionally, a definitive preoperative diagnosis might be provided from repeated cytological samplings or from several repeated biopsies [73,74]. Appropriate management for biliary G1 NETs demands surgical resection with negative margins and hepatoduodenal, periportal and pericholedochal lymphadenectomy [73,75]. Due to their low malignant potential, adequate surgical treatment leads to a favorable prognosis [76].

### 11.3. Lymphoma

About 1–2% of malignant biliary obstruction is caused by non-Hodgkin’s lymphoma (NHL), most commonly as a secondary manifestation of systemic disease. Primary non-Hodgkin’s lymphoma localized in the extrahepatic biliary ducts is extremely rare. The prevailing lymphoid neoplasm and histologic subtype of non-Hodgkin lymphoma is the diffuse large B cell lymphoma (DLBCL) [77]. Extrahepatic biliary primary NHL’s have similar features and findings with CCA in terms of imaging modalities, clinical and laboratory findings. However, in lymphomas LDH level is high and CA 19–9 level is usually normal. In terms of tumor behavior, if there is a rapid growth of the tumoral mass, it must be kept in mind that it could be lymphoma [78]. The gold standard in diagnosing NHL is pathohistological confirmation after analyzing the tissue specimen acquired by a tissue biopsy either by EUS-FNA or surgery [78]. Since the treatment is totally different in lymphomas, unnecessary surgery can be prevented upon suspicion [79].

### 11.4. Other Malignancies (Gallbladder Carcinoma, Hepatocellular Carcinoma)

Many malignant tumors that affect the hilum may mimic a CCA. A primary gallbladder cancer arising from the gallbladder neck can be difficult to differentiate from a Klatskin tumor [79].

Hepatocellular carcinoma (HCC) frequently produces a mass effect on the surrounding structures. Unlike CCA (cholangiocarcinoma), HCC rarely invades adjacent tissues. When this occurs, the diagnosis and management can be challenging and are limited to case reports [80]. 

There are reports of poorly differentiated gastric cancer with lymphatic vessel invasion and subepithelial invasion by tumor cells into the hilar bile ducts which were then pronounced to be CCA but autopsies suggested otherwise [81] as diagnosis was revealed only post-mortem.

## 12. Conclusions

**Figure 2:**
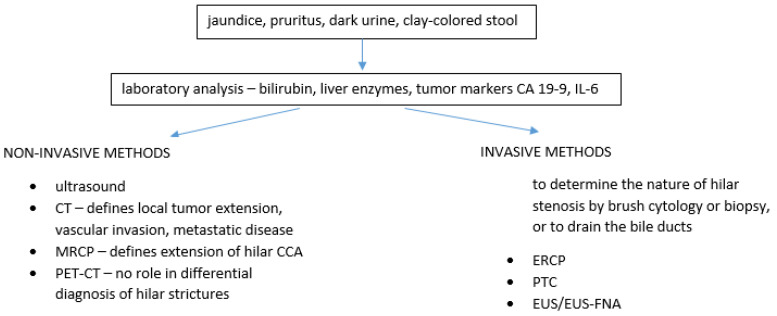
Diagnosis of hilar stenosis.

Klatskin tumor remains the most common type of CCA and carries significant morbidity and mortality. It is a diagnosis that cannot be missed, nor mistaken with any other Klatskin-like lesion, whether benign or malignant, as extensive surgery is the only curative option for CCA. This text provides a short review of other lesions that need to be taken into consideration when thinking about hilar CCA. Although imaging techniques are more powerful and detailed nowadays, making the correct diagnosis can still be quite difficult in some cases. Endoscopic methods such as ERCP with cholangioscopy and brush cytology can be of help, but also have their limitations. The clinical presentation together with available imaging, endoscopic methods and laboratory evaluation are all part of this diagnostic puzzle and a multidisciplinary approach is crucial (Figure 2).

## Figures and Tables

**Table 1:** Most common Klatskin-mimicking lesions.

**Recurrent Pyogenic Cholangitis**
**Mirizzi syndrome**
**Stricture in primary sclerosing cholangitis**
**Portal hypertensive biliopathy**
**Heterotopic tissue**
**Ischemic cholangiopathy**
**Inflammatory-infiltrative** Inflammatory pseudotumor IgG4 sclerosing cholangitis Eosinophilic cholangiopathy Mast cell cholangiopathy Follicular cholangitis Xanthogranulomatous cholangitis (XGC) Sarcoidosis
**Infective** Tuberculosis AIDS cholangiopathy Other infective causes
**Benign tumors** Extrahepatic biliary adenomas Granular cell tumors Neurofibroma Schwannoma
**Malignant tumors** Intraductal papillary neoplasms of the bile duct Neuroendocrine tumors Lymphoma Other malignancies (gallbladder carcinoma, hepatocellular carcinoma)
